# Thermodynamics and Analysis of Predicted Responses of a Phase Field Model for Ductile Fracture

**DOI:** 10.3390/ma14195842

**Published:** 2021-10-06

**Authors:** Aris Tsakmakis, Michael Vormwald

**Affiliations:** Materials Mechanics Group, Technical University Darmstadt, Franziska-Braun-Str. 3, D-64287 Darmstadt, Germany

**Keywords:** phase field, damage, plasticity, hardening, non-standard thermodynamics

## Abstract

The fundamental idea in phase field theories is to assume the presence of an additional state variable, the so-called phase field, and its gradient in the general functional used for the description of the behaviour of materials. In linear elastic fracture mechanics the phase field is employed to capture the surface energy of the crack, while in damage mechanics it represents the variable of isotropic damage. The present paper is concerned, in the context of plasticity and ductile fracture, with a commonly used phase field model in fracture mechanics. On the one hand, an appropriate framework for thermodynamical consistency is outlined. On the other hand, an analysis of the model responses for cyclic loading conditions and pure kinematic or pure isotropic hardening are shown.

## 1. Introduction

In fracture mechanics, the phase field theory has been introduced and developed in order to capture the surface energy of cracks. There have also been various attempts to extend these ideas to describe crack propagation in the case of materials exhibiting plastic material properties (see, e.g., [1,2,3,4], among others). The basic idea of phase field theories is to introduce an additional variable and its gradient in the constitutive functional modeling of the material response. Such variables are employed in physics in order to model phase transformations and the corresponding theories are known as Cahn–Hilliard theories (see, e.g., [5,6]). Generally, the gradient of the phase field variable is introduced in the theory in order to regularize the resulting field equations. In continuum damage mechanics the phase field corresponds to the isotropic damage variable and reflects, in a natural way, the physical mechanisms of crack initiation and crack propagation. The evolution of damage during the loading process causes a softening material response, rendering loss of ellipticity in the governing differential equations. Regularization by taking into account, for example, the gradient of the damage variable, is a possibility to avoid such problems [7,8]. Thus, any gradient enhanced isotropic damage theory is in principle a phase field theory (see also related remarks in [8]).

The particular advantage of damage models based on the concepts of the phase field theory, is that fracture mechanics phenomena, such as initiation, propagation, kinking and bifurcation of cracks, can be conveniently addressed in a unified manner [1]. Especially, phase field models have been applied successfully, for example, in fracture of quasi-brittle and ductile materials [1,3,4,9,10,11,12], dynamic fracture mechanics [12,13], and fatigue crack propagation [14,15,16]. The numerical benefit of the phase field method, when modeling crack propagation, is that all state variables remain continuous and the crack geometry is determined by critical values of the phase field variable. Therefore, the theory is suitable to describe equally well, both two and three dimensional problems.

A feature of special interest, when dealing with phase field models, is the appropriate thermodynamics framework. As stated above, phase field theories in fracture mechanics are nothing but gradient damage theories. Therefore, if the free energy function should depend explicitly on the phase field variable and its gradient, then thermodynamics frameworks for gradient enhanced theories in continuum mechanics will be suitable for phase field models as well. Thermodynamical concepts based on the existence of so-called microforces offer the possibility to elaborate gradients of state variables in the constitutive theory. Such ideas were introduced by Gurtin [5] and have been applied to phase field models, for example, by Borden et al. [2]. An alternative framework for gradient enhanced theories is to adopt concepts of non-conventional thermodynamics. A basic assumption in these concepts is the existence of an energy flux vector besides the standard heat flux vector (cf. [17]). Following Toupin [18], Dunn and Serrin [17] developed a non-conventional thermodynamics theory to address gradient elasticity of the Korteweg type. The main ideas of [17] are adopted in the present work to address gradient enhanced damage in plasticity. Our special interest is in a phase field law in common use, made widely known by Miehe and co-workers [9,19]. This law has been introduced primarily to model brittle fracture and is employed in several works to model ductile fracture under monotonic loading conditions. However, the question arises whether this model works equally well, when cyclic loading conditions prevail.

Thus, the paper provides both a thermodynamical and a mechanical analysis of the damage law proposed in [4,9,19]. On the one hand, consistency of the model with the governing equations of the assumed non-conventional thermodynamics is verified for the case, where the free energy function depends explicitly on the phase field variable and its gradient. On the other hand, the abilities of the model to address crack initiation and crack propagation in plasticity are reviewed. In particular, the predicted model responses in the case of cyclic loading conditions and pure kinematic or pure isotropic hardening are analysed. It is shown, that the considered model, in its basic form, is not able to describe cyclic loading programs adequately. This might be the motivation, for example, for Ulloa et al. [20] and Seles et al. [21], who introduced interesting extensions of the model, allowing them to describe loading histories involving cyclic parts. These extensions rely upon the work of Alessi et al. [16] and are briefly discussed at the end of the paper. Alternative possibilities, based on the concepts of continuum damage mechanics are also proposed.

## 2. Plasticity Coupled with Damage

A von Mises-plasticity model coupled with damage and exhibiting isotropic and kinematic hardening is assumed. All tensorial components are referred to a Cartesian coordinate system $\left\{x_{i}\right\}$. Unless stated otherwise, all indices will have the range of integers (1,2,3), while summation over repeated indices is implied. Confining on small deformations, the components $\varepsilon_{ij}$ of the strain tensor ε are denoted by

(1) $$\varepsilon_{ij}:=\frac{1}{2}(\frac{\partial u_{i}}{\partial x_{j}}+\frac{\partial u_{j}}{\partial x_{i}}),$$

where $u_{i}$ are the components of the displacement vector u. As usual, it is assumed that the additive decomposition of the strain tensor into elastic and plastic parts, $\varepsilon^{e}$ and $\varepsilon^{p}$ respectively, applies,

(2) $$\varepsilon_{ij}=\varepsilon_{ij}^{e}+\varepsilon_{ij}^{p}.$$

Let D∈[0,1] be a scalar valued damage (phase field) variable and denote by ∇D the gradient of *D*. In analogy to the concepts of continuum damage mechanics (cf., e.g., [22]), the decomposition

(3) $$\begin{array}{cc} \psi & :=\psi_{ep}+\psi_{D}=\psi_{e}+\psi_{p}^{\left(iso\right)}+\psi_{p}^{\left(kin\right)}+\psi_{D}, \end{array}$$

(4) $$\begin{array}{cc} \psi_{ep} & :=\psi_{e}+\psi_{p},\;\psi_{p}:=\psi_{p}^{\left(iso\right)}+\psi_{p}^{\left(kin\right)}. \end{array}$$

is assumed for the free energy per unit volume ψ. In metal plasticity, the parts $\psi_{e}$ and $\psi_{p}^{\left(iso\right)},\psi_{p}^{\left(kin\right)},\psi_{D}$ are responsible for the energies stored in the material due to elastic deformation of the lattice and due to distortion of the lattice caused by isotropic hardening, kinematic hardening and damage evolution, respectively. There are some characteristic features with regard to the form of ψ. First, a common assumption is, that $\psi_{e}=\psi_{e}\left(\varepsilon^{e},D\right)$. Often, an additive decomposition of $\psi_{e}$ into tensile and compressive contributions $\psi_{e}^{+}$ and $\psi_{e}^{-}$ is adopted. A possibility advocated, for example, by Miehe et al. [9], is to relate $\psi_{e}^{+}$ and $\psi_{e}^{-}$ to the sign of the principal strains. This approach will not be considered here, because of numerical instabilities and convergence problems in the case of plasticity (cf. also related remarks in [2]). Another possibility proposed by Amor et al. [23] and used in the current paper, is based on a volumetric-deviatoric split and can be expressed in the form

(5) $$\begin{array}{cc} \psi_{e} & =\psi_{e}\left(\varepsilon^{e},D\right):=\psi_{e}^{+}\left(\varepsilon^{e},D\right)+\psi_{e}^{-}\left(\varepsilon^{e}\right), \end{array}$$

(6) $$\begin{array}{cc} \psi_{e}^{+} & =\psi_{e}^{+}\left(\varepsilon^{e},D\right):=g\left(D\right)\psi_{e}^{0+}=g\left(D\right)\{\frac{1}{2}K{\langle \varepsilon_{kk}^{e}\rangle }^{2}+\mu {\left(\varepsilon_{ij}^{e}\right)}^{\mathrm{dev}}{\left(\varepsilon_{ij}^{e}\right)}^{\mathrm{dev}}\}, \end{array}$$

(7) $$\begin{array}{cc} \psi_{e}^{-} & =\psi_{e}^{-}\left(\varepsilon^{e}\right):=\frac{1}{2}K{\langle -\varepsilon_{kk}^{e}\rangle }^{2}, \end{array}$$

where *K* is the compression modulus, μ is the shear modulus, g(D) is a scalar valued degradation function and $\langle x\rangle :=\frac{1}{2}\left(x+|x|\right)$. Tensile and compressive contributions to the elastic part of the free energy are distinguished on the basis of the sign of the trace of the elastic strain tensor. Damage accumulation affects only the tensile part by reduction of the elastic stiffness through g(D). A common assumption for the degradation function is

(8) $$g\left(D\right)={\left(1-D\right)}^{2}+\kappa ,$$

where κ≪1 is a parameter for numerical stability. In Equation (6) and in the following, undegradated parts of the free energy are denoted with the superscript 0. The elasticity law for the Cauchy stress σ may then be viewed as defined by the potential relation

(9) $$\sigma_{ij}=\frac{\partial \psi }{\partial \varepsilon_{ij}^{e}}=g\left(D\right)\frac{\partial \psi_{e}^{0+}}{\partial \varepsilon_{ij}^{e}}+\frac{\partial \psi_{e}^{0-}}{\partial \varepsilon_{ij}^{e}}=K\left[g\left(D\right)\langle \varepsilon_{kk}^{e}\rangle -\langle -\varepsilon_{kk}^{e}\rangle \right]\delta_{ij}+g\left(D\right)2\mu {\left(\varepsilon_{ij}^{e}\right)}^{\mathrm{dev}},$$

where $\delta_{ij}$ is the Kronecker-delta symbol and $A^{\mathrm{dev}}$ is the deviator of the second-order tensor A.

Another characteristic feature concerns the form of $\psi_{p}$, which may or may not depend on the damage variable *D*. Generally, in continuum damage mechanics the assumed form of $\psi_{p}$ is closely related to the assumed form of the yield function. There are several concepts for deriving the form of the part $\psi_{p}$ and the form of the yield function from corresponding plasticity models without damage mechanisms. These concepts are based on the principles of stress, strain or energy equivalence (see, e.g., [22,24,25,26,27]). Here, we adopt the formulations proposed in Grammenoudis et al. [27], which are based on a specific version of the principle of energy equivalence. Moreover, for the purpose of the present work, it suffices to confine to linear isotropic and linear kinematic hardenings. Hence,

(10) $$\begin{array}{cc} \; & \psi_{p}=\psi_{p}\left(s,\varepsilon^{p},D\right)=g\left(D\right)\psi_{p}^{0}\left(s,\varepsilon^{p}\right)=g\left(D\right)\psi_{p}^{0(iso)}+g\left(D\right)\psi_{p}^{0(kin)}, \end{array}$$

(11) $$\begin{array}{cc} \; & \psi_{p}^{0(iso)}=\psi_{p}^{0(iso)}\left(s\right):=\frac{1}{2}\gamma s^{2}, \end{array}$$

(12) $$\begin{array}{cc} \; & \psi_{p}^{0(kin)}=\psi_{p}^{0(kin)}\left(\varepsilon^{p}\right):=\frac{1}{2}c\varepsilon_{ij}^{p}\varepsilon_{ij}^{p}, \end{array}$$

(13) $$\begin{array}{cc} \; & \psi_{ep}^{0+}:=\psi_{e}^{0+}+\psi_{p}^{0(iso)}+\psi_{p}^{0(kin)}, \end{array}$$

where γ,c are the respective hardening coefficients. On defining by $\dot{\left(\cdot \right)}$ the derivative of (·) with respect to time *t*, the plastic arc length *s* is given by

(14) $$\dot{s}:=\sqrt{\frac{2}{3}{\dot{\varepsilon }}_{ij}^{p}{\dot{\varepsilon }}_{ij}^{p}}.$$

Scalar internal stress *R* reflecting isotropic hardening and the backstress tensor ξ of kinematic hardening are given by the potential relations

(15) $$\begin{array}{cc} R & =R\left(s,D\right)=\frac{\partial \psi }{\partial s}=g\left(D\right)\gamma s, \end{array}$$

(16) $$\begin{array}{cc} \xi_{ij} & =\xi_{ij}\left(\varepsilon^{p},D\right)=\frac{\partial \psi }{\partial \varepsilon_{ij}^{p}}=g\left(D\right)c\varepsilon_{ij}^{p}. \end{array}$$

A generalization of the von Mises-yield function reads in [27]

(17) $$\begin{array}{cc} F & =F\left(\sigma ,R,\xi ,D\right):=g_{f}\left(D\right)f\left(\sigma ,\xi ,R\right)-k_{0}, \end{array}$$

(18) $$\begin{array}{cc} f & :=\sqrt{\frac{3}{2}{\left(\sigma_{ij}-\xi_{ij}\right)}^{\mathrm{dev}}{\left(\sigma_{ij}-\xi_{ij}\right)}^{\mathrm{dev}}}-R. \end{array}$$

In this equation, $k_{0}$ is a material parameter representing the initial yield stress and $g_{f}\left(D\right)$ is a further degradation function capturing damage mechanisms during plastic flow. Here,

(19) $$g_{f}\left(D\right)=g^{-1}\left(D\right)$$

is chosen, so that the yield function in Equation (17) is the same as in Borden et al. [2], Kuhn et al. [3] and Huang et al. [28]. Other possibilities for the function $g_{f}$ are discussed in Reckwerth et al. [29]. For the evolution law of plastic strain, an associated normality rule is assumed,

(20) $${\dot{\varepsilon }}_{ij}^{p}=\Lambda \frac{\partial F}{\partial \sigma_{ij}},$$

with Λ denoting a scalar plastic multiplier, together with the Kuhn–Tucker conditions (cf. [30,31]),

(21) Λ≥0,F≤0,ΛF=0,

and the consistency condition, that during plastic flow

(22) $$\Lambda \geq 0,\;\dot{F}\leq 0,\;\Lambda \dot{F}=0.$$

A further characteristic feature of interest refers to the term $\psi_{D}$. Some works (see, e.g., [4,9,19]), dealing with classical thermodynamics, assume a vanishing part $\psi_{D}$ and incorporate ∇D in the postulated damage criterion and dissipation function. Other approaches, pursued, for example, by Borden et al. [2], admit the existence of $\psi_{D}$ and assume it to depend on *D* and ∇D. Such works deal with classical thermodynamics, but postulate the existence of microforces and related balance laws, in order to render the constitutive theory thermodynamically consistent. The microforces approach has been employed by Gurtin in order to establish equations of the Ginzburg–Landau and Cahn–Hilliard type [5]. Note, that an evolution equation of the Ginzburg–Landau type for the damage variable in plasticity coupled with damage has been supposed in the works [3,10,32]. Generally, when gradients of state variables are present in the response function for ψ, classical thermodynamics, dealing only with classical forces, is not an appropriate framework. Whenever $\psi_{D}$, and therefore ψ too, depend on ∇D, an alternative to the approach based on microforces, in order to achieve thermodynamical consistency, is provided by non-conventional thermodynamics frameworks. In the next section, the non-conventional thermodynamics proposed by Dunn and Serrin [17] are applied in order to model gradient damage mechanisms. The obtained results rely upon the ansatz

(23) $$\psi_{D}=\psi_{D}\left(D,\nabla D\right)=G_{c}(\frac{1}{2l}D^{2}+\frac{l}{2}{\left|\nabla D\right|}^{2}),$$

which is standard in this subject matter (cf., e.g., [1,2,10,28,33]). In this equation, $G_{c}$ and *l* are material parameters, with *l* denoting a material internal length.

## 3. Thermodynamical Formulation

### 3.1. Non-Conventional Thermodynamics

Let *V* be the range in the three dimensional Euclidean point space occupied by a material body B, with boundary ∂V, and denote by n the outward unit vector on ∂V. The location vector to material points in V∪∂V is denoted by x with components $x_{i}$. In standard thermodynamics, the energy balance law is expressed in terms of the heat flux vector $\bar{q}$. For the aims of the present work, and following the suggestions by Toupin [18] and Dunn and Serrin [17], the conventional form of the energy balance law is generalized by admitting the existence of an energy flux vector $q^{\prime }$, besides the heat flux vector $\bar{q}$. Thus, omitting acceleration terms, body forces and heat supply, and denoting by *e* the internal energy per unit volume, the global form of the energy balance law reads

(24) $$\frac{d}{dt}\int_{V}e\;dV=\int_{\partial V}n_{i}\sigma_{ij}{\dot{u}}_{j}\;dA-\int_{\partial V}q_{i}^{\prime }n_{i}\;dA-\int_{\partial V}{\bar{q}}_{i}n_{i}\;dA.$$

After localization, and keeping in mind the definition of strain in Equation (1), the local form of the energy balance

(25) $$\dot{e}=\sigma_{ij}{\dot{\varepsilon }}_{ij}-\partial_{i}q_{i}^{\prime }-\partial_{i}{\bar{q}}_{i}$$

is obtained, where $\partial_{i}\left(\;\right)=\partial \left(\;\right)/\partial x_{i}$. The energy carriers responsible for $q^{\prime }$ in the cases of gradient elasticity, gradient plasticity and gradient damage mechanisms may be viewed to be related to interstitials, dislocations and initiation and evolution of damage defects, respectively.

Let θ>0 be the absolute temperature, η the entropy per unit volume and ψ, as above, the free energy per unit volume, so that the Legendre transformation

(26) e=ψ+θη

applies. For general thermomechanical processes, the constitutive theory dealt with, is characterized by a free energy of the form

(27) $$\psi =\psi (\varepsilon^{e},s,\varepsilon^{p},D,\nabla D,\theta ).$$

It follows from Equations (25) and (26), that

(28) $$\sigma_{ij}{\dot{\varepsilon }}_{ij}-\dot{\psi }-\theta \dot{\eta }-\dot{\theta }\eta -\partial_{i}q_{i}^{\prime }-\partial_{i}{\bar{q}}_{i}=0.$$

Further, the validity of the Clausius–Duhem inequality in the local form is assumed (cf. [17])

(29) $$\dot{\eta }+\partial_{i}\left(\frac{{\bar{q}}_{i}}{\theta }\right)\geq 0,$$

or equivalently, by virtue of Equation (28),

(30) $$\sigma_{ij}{\dot{\varepsilon }}_{ij}-\partial_{i}q_{i}^{\prime }-\dot{\psi }-\eta \dot{\theta }-\frac{1}{\theta }{\bar{q}}_{i}\partial_{i}\theta \geq 0.$$

In the next section, the response function for the energy flux vector $q^{\prime }$ is specified.

### 3.2. Dissipation Inequality

Equations (2) and (27) are now inserted into Equation (30), to obtain

(31) $$\sigma_{ij}{\dot{\varepsilon }}_{ij}-\partial_{i}q_{i}^{\prime }-\left[\frac{\partial \psi }{\partial \varepsilon_{ij}^{e}}{\dot{\varepsilon }}_{ij}-\frac{\partial \psi }{\partial \varepsilon_{ij}^{e}}{\dot{\varepsilon }}_{ij}^{p}+\frac{\partial \psi }{\partial s}\dot{s}+\frac{\partial \psi }{\partial \varepsilon_{ij}^{p}}{\dot{\varepsilon }}_{ij}^{p}+\frac{\partial \psi }{\partial D}\dot{D}+\frac{\partial \psi }{\partial {\left(\nabla D\right)}_{i}}{\left(\nabla \dot{D}\right)}_{i}+\frac{\partial \psi }{\partial \theta }\dot{\theta }\right]-\eta \dot{\theta }-\frac{1}{\theta }{\bar{q}}_{i}\partial_{i}\theta \geq 0.$$

Using standard arguments, it can be deduced from this inequality, that

(32) $$\begin{array}{c} \sigma_{ij}=\frac{\partial \psi (\varepsilon^{e},s,\varepsilon^{p},D,\nabla D,\theta )}{\partial \varepsilon_{ij}^{e}}, \end{array}$$

(33) $$\begin{array}{c} \eta =-\frac{\partial \psi (\varepsilon^{e},s,\varepsilon^{p},D,\nabla D,\theta )}{\partial \theta }, \end{array}$$

and that

(34) $$-\partial_{i}q_{i}^{\prime }-\left[-\sigma_{ij}{\dot{\varepsilon }}_{ij}^{p}+\frac{\partial \psi }{\partial s}\dot{s}+\frac{\partial \psi }{\partial \varepsilon_{ij}^{p}}{\dot{\varepsilon }}_{ij}^{p}+\frac{\partial \psi }{\partial D}\dot{D}+\frac{\partial \psi }{\partial {\left(\nabla D\right)}_{i}}{\left(\nabla \dot{D}\right)}_{i}\right]-\frac{1}{\theta }{\bar{q}}_{i}\partial_{i}\theta \geq 0.$$

As usual, the sufficient conditions

(35) $$\begin{array}{c} -\partial_{i}q_{i}^{\prime }-\left[-\sigma_{ij}{\dot{\varepsilon }}_{ij}^{p}+\frac{\partial \psi }{\partial s}\dot{s}+\frac{\partial \psi }{\partial \varepsilon_{ij}^{p}}{\dot{\varepsilon }}_{ij}^{p}+\frac{\partial \psi }{\partial D}\dot{D}+\frac{\partial \psi }{\partial {\left(\nabla D\right)}_{i}}{\left(\nabla \dot{D}\right)}_{i}\right]\geq 0, \end{array}$$

(36) $$\begin{array}{c} -\frac{1}{\theta }{\bar{q}}_{i}\partial_{i}\theta \geq 0, \end{array}$$

are assumed for Equation (34) to apply. Equation (35) is called the intrinsic dissipation inequality. In the remainder of the paper, isothermal deformations with uniformly distributed temperature are supposed to apply, so that θ can be omitted in the response functions. Then, by assuming ψ to be given as in Section 2, so that $\sigma_{ij}$ in Equation (32) is given by the elasticity law (9), and making use of the potential relations for ξ,R introduced in Equations (15) and (16), Equation (35) becomes

(37) $$-\partial_{i}q_{i}^{\prime }+\left(\sigma_{ij}-\xi_{ij}\right){\dot{\varepsilon }}_{ij}^{p}-R\dot{s}-\frac{\delta \psi }{\delta D}\dot{D}-\frac{\partial }{\partial x_{i}}\left(\frac{\partial \psi }{\partial {\left(\nabla D\right)}_{i}}\dot{D}\right)\geq 0.$$

Here, the variational derivative δψ/δD is defined through

(38) $$\frac{\delta \psi }{\delta D}:=\frac{\partial \psi }{\partial D}-\frac{\partial }{\partial x_{i}}\left(\frac{\partial \psi }{\partial {\left(\nabla D\right)}_{i}}\right).$$

During plastic flow

(39) $$F=0\Leftrightarrow \sqrt{\frac{3}{2}{\left(\sigma_{ij}-\xi_{ij}\right)}^{\mathrm{dev}}{\left(\sigma_{ij}-\xi_{ij}\right)}^{\mathrm{dev}}}=R+\frac{k_{0}}{g_{f}\left(D\right)},$$

so that evolution Equation (20) can be written in the form

(40) $${\dot{\varepsilon }}_{ij}^{p}=\frac{3}{2}\Lambda g_{f}\left(D\right)\frac{{\left(\sigma_{ij}-\xi_{ij}\right)}^{\mathrm{dev}}}{R+k_{0}/g_{f}\left(D\right)},$$

from which $\dot{s}=\Lambda g_{f}\left(D\right)$. It follows, that

(41) $$\left(\sigma_{ij}-\xi_{ij}\right){\dot{\varepsilon }}_{ij}^{p}={\left(\sigma_{ij}-\xi_{ij}\right)}^{\mathrm{dev}}{\dot{\varepsilon }}_{ij}^{p}=(R+\frac{k_{0}}{g_{f}\left(D\right)})\dot{s},$$

or

(42) $$\left(\sigma_{ij}-\xi_{ij}\right){\dot{\varepsilon }}_{ij}^{p}-R\dot{s}=\frac{k_{0}}{g_{f}\left(D\right)}\dot{s}\geq 0$$

provided $g_{f}\left(D\right),k_{0}\geq 0$. Therefore, it suffices to require

(43) $$-\partial_{i}q_{i}^{\prime }-\frac{\delta \psi }{\delta D}\dot{D}-\frac{\partial }{\partial x_{i}}(\frac{\partial \psi }{\partial {\left(\nabla D\right)}_{i}}\dot{D})\geq 0,$$

in order to satisfy Equation (37). The simplest possibility to always fulfil this inequality is to make the constitutive assumption

(44) $$q_{i}^{\prime }=-\frac{\partial \psi }{\partial {\left(\nabla D\right)}_{i}}\dot{D}+c_{i}=-G_{c}l{\left(\nabla D\right)}_{i}\dot{D}+c_{i},$$

where $c_{i}$ are the components of a divergence-free vector c. For reasons of simplicity, c is assumed to vanish in the following. This way, Equation (43) reduces to

(45) $$\Omega \dot{D}\geq 0,$$

where

(46) $$\Omega :=-\frac{\delta \psi }{\delta D}=-\frac{\partial \psi_{ep}}{\partial D}-\frac{\delta \psi_{D}}{\delta D}.$$

Before closing this section, it should be mentioned, that Maugin [34] also derived Equation (45) without assuming the existence of an energy flux vector in the energy balance law. His theory is based on a form of the second law proposed by Müller [35], which introduces an extra entropy flux term in the Clausius–Duhem inequality, besides the classical one. Therefore, for general thermomechanical processes, the two approaches are different.

## 4. The Damage Law of Miehe and Co-Workers

The aim of this section is to prove consistency with the adopted non-conventional thermodynamics of a damage law in common use, which has been proposed by Miehe and co-workers (see, e.g., [19]).

It can be recognized from Equation (45), that Ω is the driving force for damage evolution. Therefore, in analogy to plasticity and in order to always fulfil Equation (45), the existence of a damage function $F_{D}\leq 0$ of Ω is admitted with the assumption that the set of Ω-values with $F_{D}\leq 0$ includes Ω=0. Damage evolution takes place only when the damage condition $F_{D}=0$ holds. Additionally, $\dot{D}$ is set to be directed along the outward normal to the level set of $F_{D}$, $\dot{D}=\Lambda_{D}\partial F_{D}/\partial \Omega$, where $F_{D},\Lambda_{D}$ are subject to the Kuhn–Tucker conditions

(47) $$\Lambda_{D}\geq 0,\;F_{D}\leq 0,\;\Lambda_{D}F_{D}=0,$$

and the consistency condition during damage evolution

(48) $$\Lambda_{D}\geq 0,\;{\dot{F}}_{D}\leq 0,\;\Lambda_{D}{\dot{F}}_{D}=0$$

(cf. Equations (21) and (22)). A simple form for $F_{D}$ reads

(49) $$F_{D}:=\Omega -k_{D}\leq 0,$$

where $k_{D}$ is the analog of the yield stress in the yield function and can depend on the material state. Aifantis [36] proposed to assume the yield stress in gradient plasticity as a function of the plastic arc length *s* and its spatial derivative Δs, where Δ is the Laplace operator. In its linear form, and when the initial yield stress $k_{0}$ vanishes, this function reads

(50) R=γs−αΔs

and represents isotropic hardening, where γ is defined as in Equation (15) and α is a further non-negative material parameter (cf. also Section 89 in [37]). In the damage model, *D* is considered to be the counterpart of *s* in plasticity. Further, it is assumed, that $k_{D}$ in Equation (49) does not include a constant threshold and it is remarked from Equation (23), that

(51) $$\frac{\delta \psi_{D}}{\delta D}=\frac{G_{c}}{l}(D-l^{2}\Delta D).$$

A comparison of the latter with Equation (50) suggests to set

(52) $$k_{D}=\beta \frac{\delta \psi_{D}}{\delta D},$$

with β being a non-negative parameter. It follows from Equations (46), (49) and (52), that

(53) $$-\frac{\partial \psi_{ep}}{\partial D}\leq \left(\beta +1\right)\frac{\delta \psi_{D}}{\delta D}.$$

Then, from Equations (4)–(8), (10) and (13), we have

(54) $$-\frac{\partial \psi_{ep}}{\partial D}=2\left(1-D\right)\psi_{ep}^{0+}=2\left(1-D\right)(\psi_{e}^{0+}+\psi_{p}^{0(iso)}+\psi_{p}^{0(kin)})\geq 0,$$

and by virtue of Equation (53),

(55) $$\frac{\delta \psi_{D}}{\delta D}\geq 0.$$

Because of the latter, the sufficient and necessary condition for Equation (45) is $\dot{D}\geq 0$, which means that damage can only increase and that healing processes are excluded. In fact, from Equations (47)–(49), we have

(56) $$\dot{D}=\Lambda_{D}\geq 0,$$

and hence Equation (45) is always satisfied. The two Equations (53) and (56) are essentially the damage law proposed in Miehe et al. [19]. It is readily seen, from Equations (51), (53) and (54), that during damage evolution

(57) $$2\left(1-D\right)\psi_{ep}^{0+}-\left(\beta +1\right)\frac{G_{c}}{l}\left(D-l^{2}\Delta D\right)=0.$$

Clearly, during damage evolution $\psi_{ep}^{0+}$ is a monotonically increasing function of time and thus, following Miehe et al. [19], it is convenient to define the history variable

(58) $$H\left(x,t\right):=\max_{\tau \in [0,t]}\psi_{ep}^{0+}\left(x,\tau \right).$$

Hence, the governing partial differential equation to be solved for the phase field problem reads

(59) $$2\left(1-D\right)H-\left(\beta +1\right)\frac{G_{c}}{l}\left(D-l^{2}\Delta D\right)=0.$$

Section 6 provides an analysis of the damage model with reference to one- and two-dimensional examples.

## 5. Finite Element Implementation

The numerical integration of the constitutive theory presented in the previous sections is performed in a finite-element framework, with the damage variable being treated as an additional degree of freedom at every node. A staggered algorithm, as proposed in Miehe et al. [19], is implemented in the commercial software package ABAQUS. Within a time increment, the displacement problem is solved first, while the damage variable is held constant. In a second step, the phase field problem is solved, while the displacement is held constant. A user material subroutine (UMAT) has been developed for the displacement problem, which is based on the method of elastic predictor and plastic corrector, cf. Simo and Hughes [30]. The required consistent tangent operator is calculated by numerical differentiation. The solution of the phase field problem is based on a weak form of the partial differential equation Equation (59), see, for example [21,38]. The discretized form of the resulting formulation was incorporated in a user element subroutine (UEL). The advantage of the staggered algorithm is its great robustness. This is of particular interest, since the deformations in the vicinity of the crack tip are very high, which can lead to convergence problems in the context of elastoplastic material models.

For the examples discussed in the next section, linear shape functions for both the displacement and the phase field problem are used. All material parameters are listed in Table 1. Note, that the material parameters in the phase field model are the same as in [9].

## 6. Analysis of Predicted Responses

**Figure 1:**
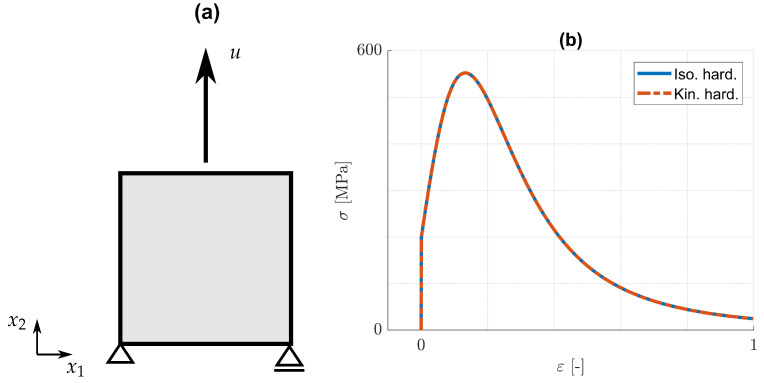
(**a**) One-dimensional model. Eight-node element subjected to tension/compression loading along the $x_{2}$-axis. (**b**) Identical (ε,σ)-distributions due to pure isotropic and pure kinematic hardening for monotonic loading conditions.

**Figure 2:**
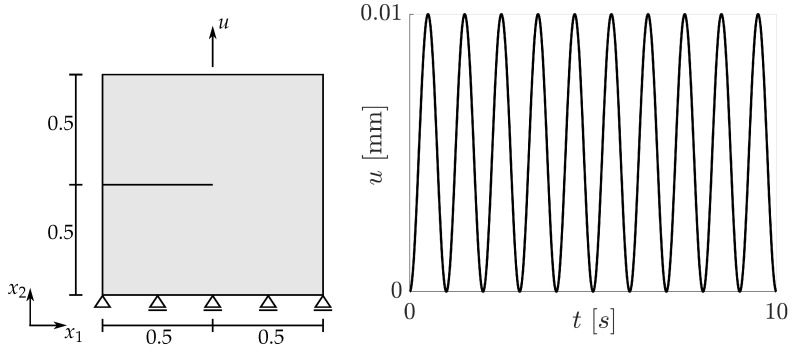
Geometry and loading history for the cracked specimen. The specimen is discretized by 6684 linear, four-node plane strain elements. All dimensions are in mm.

It is of interest now to analyse the effect of the damage model on the predicted responses. For the aims of the present paper, as mentioned in the introduction, it suffices to confine the analysis to cyclic loading conditions for the cases of pure kinematic or pure isotropic hardening. The discussions rely upon one- and two-dimensional examples. The one-dimensional examples refer to an eight-node element, cf. Figure 1, while the two-dimensional examples concern the cracked specimen shown in Figure 2. In the latter, linear four-node plane strain elements (CPE4) are used for the displacement problem. In all cases, homogeneous Neumann boundary conditions are supposed to apply for the phase field problem. In order to facilitate comparison of the results, the material parameters for isotropic and kinematic hardening are chosen in the form γ=3c/2, so that the predicted strain–stress distributions for one-dimensional monotonic loading are identical. This is demonstrated in Figure 1b, where $\sigma =\sigma_{22}$ and $\varepsilon =\varepsilon_{22}$ are the stress and strain components in the loading direction. Note that only the form of the strain–stress curve in Figure 1b, which is a characteristic feature of the assumptions made, is of interest. Moreover, such distributions as (ε,σ) indicate graphs of points (ε(t),σ(t)) parametrized by time *t*. In both, the one- and the two-dimensional examples, the top boundary is subjected to a prescribed displacement, while all other boundary conditions are as shown in Figure 1 and Figure 2. The imposed displacement in the one-dimensional case varies harmonically with vanishing mean value. This corresponds to strain-controlled, homogeneous tension/compression loading between two bounding strains $-\varepsilon_{0}$ and $\varepsilon_{0}$. The cracked specimen is also subjected to harmonically varying displacement, but with positive mean value, cf. Figure 2.

**Figure 3:**
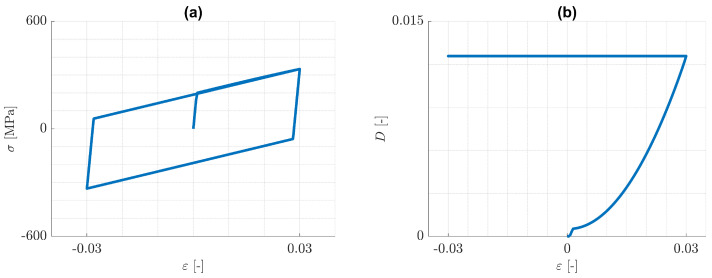
Cyclic, uniaxial tension/compression loading: pure kinematic hardening. Predicted (**a**) (ε,σ)-distribution and (**b**) (ε,D)-distribution.

First, pure kinematic hardening in the one-dimensional case is considered. From the predicted (ε,σ)-distributions displayed in Figure 3a, it can be recognized, that the material response reduces to a closed hysteresis loop just after one loading cycle. This behaviour is quite similar to the case of cyclic plasticity without damage and arises from the fact, that damage evolution is involved only in the first tension loading branch (see Figure 3b). It becomes clear from Equation (59), that damage evolution can only occur, if the value of the history variable H increases. In the present case, the maximum value of H, and hence of the damage variable *D* too, is obtained at the end of the first tension loading branch. After that, both H and *D* always remain constant for this model. As a consequence, the split in the elasticity law in Equation (9) has a negligible effect and the maximal amounts of the plastic strains in both tension and compression, remain constant and practically equal to each other.

**Figure 4:**
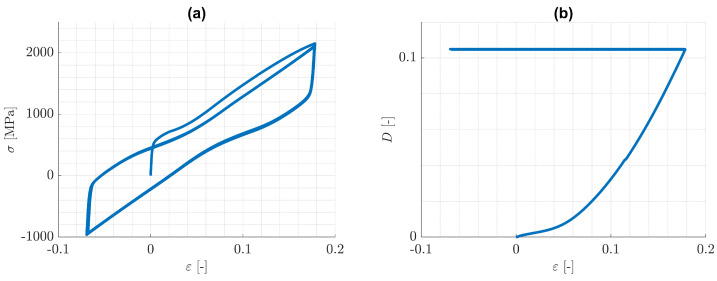
(**a**) $\left(\varepsilon_{22},\sigma_{22}\right)$-distribution and (**b**) (ε,D)-distribution for the first integration point behind the crack tip.

**Figure 5:**
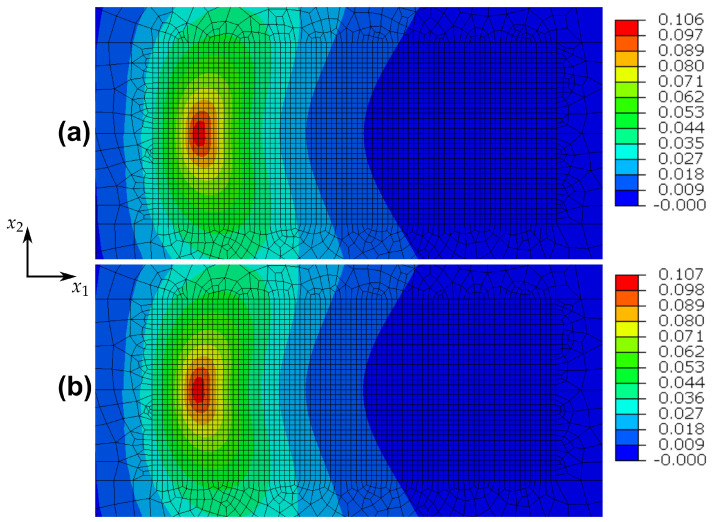
Pure kinematic hardening: damage evolution for the cracked specimen after (**a**) one loading cycle and after (**b**) ten loading cycles.

These issues for one-dimensional homogeneous deformations are somewhat similar in the case of the cracked specimen indicated in Figure 2. To elucidate, the nearest integration point behind the crack tip is considered. The predicted $\left(\varepsilon_{22},\sigma_{22}\right)$- and (ε,D)-distributions for this point are shown in Figure 4a,b and reveal, that after the first tension loading branch, the damage value remains practically constant, whereas the plastic strain is changing in every loading cycle. The damage distribution for the whole specimen after one and after ten loading cycles is shown in Figure 5a,b, where the respective maximum values of damage are depicted in a red colour. It is obvious, that damage does not accumulate and therefore the model fails to describe fatigue crack propagation. These results make clear, that in general, the adopted phase field theory is not qualified to address ductile fracture, when only kinematic hardening is present.

**Figure 6:**
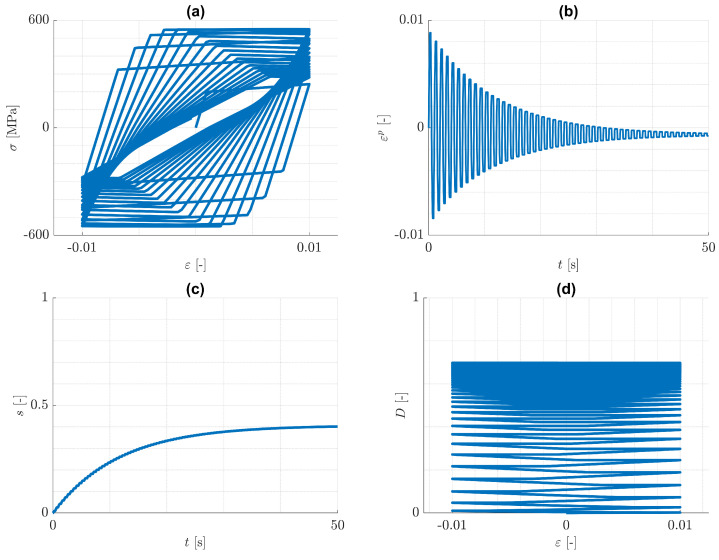
Pure isotropic hardening: cyclic, uniaxial tension/compression loading with $\varepsilon_{0}=0,01$. Predicted (**a**) (ε,σ)-distribution, (**b**) $\left(t,\varepsilon^{p}\right)$-distribution, (**c**) (t,s)-distribution and (**d**) (ε,D)-distribution.

**Figure 7:**
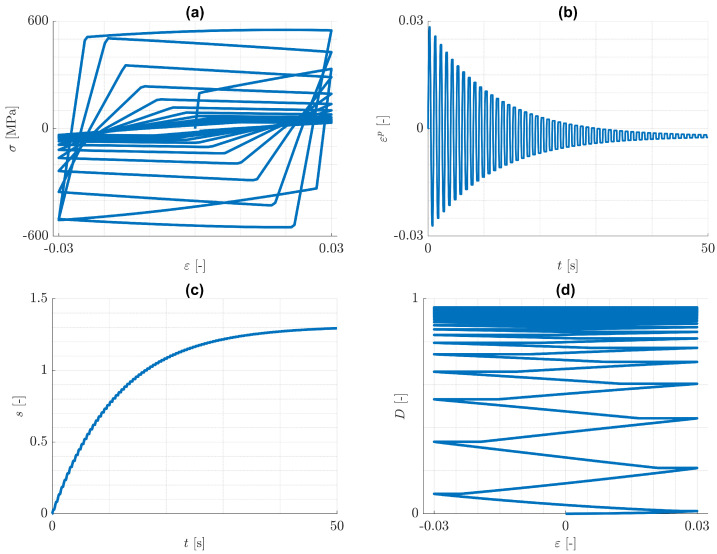
Pure isotropic hardening: cyclic, uniaxial tension/compression loading with $\varepsilon_{0}=0,03$. Predicted (**a**) (ε,σ)-distribution, (**b**) $\left(t,\varepsilon^{p}\right)$-distribution, (**c**) (t,s)-distribution and (**d**) (ε,D)-distribution.

**Figure 8:**
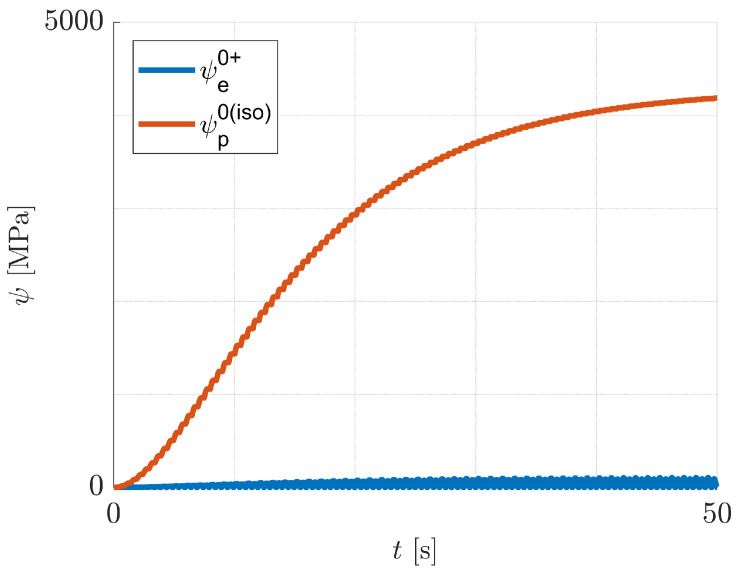
Pure isotropic hardening: evolution of $\psi_{e}^{0+}$ and $\psi_{p}^{0(iso)}$ with time *t* for $\varepsilon_{0}=0.03$.

Next, the case of pure isotropic hardening is discussed. Predicted responses for the imposed one-dimensional cyclic loading conditions are illustrated in Figure 6 and Figure 7, where $\varepsilon^{p}=\varepsilon_{11}^{p}$. It can be seen from Figure 6d and Figure 7d, that the increase of damage is practically equal for tension and for compression. The reason for this behaviour is that the history variable H in Equation (59) is dominated by $\psi_{p}^{0(iso)}$, which increases practically equally in both tension and compression, see Figure 8. That means, the tension/compression asymmetry in the elasticity law has a minor influence on the damage model under consideration.

It can be seen from Figure 6b and Figure 7b, that with increasing number of loading cycles, the amount of plastic strain decreases and approaches to a constant value. This is a consequence of the assumption, that the mean value of the strain disappears. At the same time, the plastic arc length *s* and the damage variable *D* approach to limits $s^{*}$ and $D^{*}$, each of which is a monotonically increasing function of $\varepsilon_{0}$. This behaviour is again similar to the case of cyclic plasticity without damage. Actually, it can be verified for cyclic plasticity without damage, that the yield radius approaches a limiting value $k_{0}+\gamma s^{*}$. The value $s^{*}$ can be estimated from the yield condition and the elasticity law to be $s^{*}=\left(E\varepsilon_{0}-k_{0}\right)/\gamma$, where *E* is the Young’s modulus. It is worth remarking, that opposite to the case of plasticity without damage, there is a limiting constant value of plastic strain, which is negative. This is an implication of both the split in the elasticity law in Equation (9), that now has a noticeable influence, and the isotropic hardening, that changes in every cycle. It is concluded, that for the considered one-dimensional problems, similarly to the case of pure kinematic hardening, damage accumulation can be bounded by values smaller than one.

**Figure 9:**
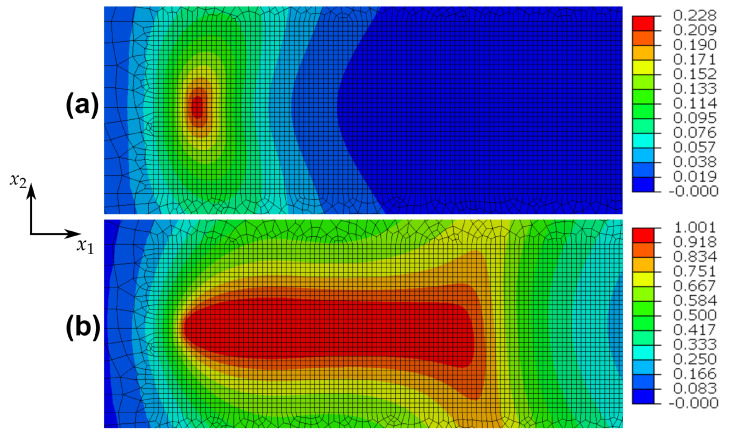
Pure isotropic hardening: damage evolution for the cracked specimen after (**a**) one loading cycle and after (**b**) ten loading cycles.

Opposite to pure kinematic hardening, these conclusions do not hold for the structural problem of the cracked specimen. Since the amount of the local strains are not subjected to constraints, damage accumulates continuously in the vicinity of the crack tip and approaches 1. Figure 9a,b illustrates the damage distribution after one and after ten loading cycles and makes clear, that the range with values of *D* close to 1 becomes larger with increasing number of loading cycles. Consequently, a description of fatigue crack propagation is possible in principle. However, the following should be remarked. It is well known, that linear isotropic hardening cannot capture adequately effects of cyclic plasticity. Furthermore, if non-linear isotropic hardening is assumed, so that *R* is bounded from above, then this model does not permit D→1 even for monotonic, homogeneous loading. This assertion can be proved on the basis of Equation (59), from which

(60) $$D=\frac{2H}{2H+\frac{\left(1+\beta \right)G_{c}}{l}}.$$

Evidently, D→1 only when H→∞, which cannot happen, as $\psi_{ep}^{0+}$, and hence H too, are bounded for this case.

## 7. Concluding Remarks

The present paper provides an analysis of a phase field model in common use. The analysis comprises thermodynamical aspects and characteristic features concerning ductile fracture mechanics. It is shown that, if the free energy function depends explicitly on D,∇D, then thermodynamical consistency of the phase field model can be well addressed in the framework of non-conventional thermodynamics. The basic structure of the constitutive theory is adopted from phenomenological plasticity combined with continuum damage mechanics methods. For the sake of simplicity, only pure kinematic or pure isotropic hardening are incorporated. It is shown, with reference to cyclic loading conditions, that the phase field model under consideration, in its basic form, is not able to address ductile fracture mechanics problems. A further characteristic feature is that tension/compression asymmetry is modeled in the elastic part of the free energy function and cannot be controlled separately by material parameters during plastic loading.

The results of this or similar analyses were certainly known, for example, to the authors of the papers Ulloa et al. [20] and Seles et al. [21]. Therefore, as mentioned in the introduction, based on an idea developed for the first time in Alessi et al. [16], these authors proposed extensions of the basic structure of the model by introducing a further degradation function, depending on a so-called fatigue variable. It is worth noting, that like the basic form of the model discussed in the present paper, the fatigue generalizations of the model are rather extended models of fracture mechanics. As such, they originate from the regularization of sharp crack topologies, where the relevant crack propagation mechanism is based on the debonding of atomic planes. Therefore, both the formulations in Miehe and co-workers [4,9,19], as well as the mentioned fatigue extensions, only consider degradation of the material stiffnesses in the free energy. Energy stored in the material due to the damage process, as modeled by the part $\psi_{D}$ in the present paper, is not intended. Note also, that cyclic loading effects are reflected in the work of Alessi et al. [16] by degradation of the fracture toughness depending on the accumulated plastic strain. It should be outlined, however, that the present forms of these extensions deal with evolution equations of the damage variable that do not account for plastic rate effects.

An alternative to such approaches arises, if the analysis above is interpreted to suggest modeling of the constitutive response of ductile materials within the context of continuum damage mechanics. Accordingly, the failure process of, for example, metallic materials, has to be viewed as the result of initiation, growth and coalescence of voids. Opposite to brittle materials, local distortion of the lattice due to the existence of voids will now contribute to the energy stored in the material in terms of the part $\psi_{D}$, besides the energy stored due to the elastic deformation of the lattice and distortion of the lattice due to the creation and motion of dislocations, leading to hardening effects. The analysis of the present paper demonstrates, that non-conventional thermodynamics is an appropriate framework for free energy functions of such forms. Further, according to the methods of continuum damage mechanics, the evolution equation of *D* should be related to the rate of the plastic arc length $\dot{s}$. A common simplification is to regard the tension/compression asymmetry to be relevant only for the damage law. In this case, the asymmetry can be reflected by the damage potential on which the damage evolution is based (see, e.g., Malcher and Mamyia [39] and the references cited there). This way, tension/compression asymmetry aspects can be controlled by material parameters. The thermodynamics adopted can address such issues appropriately as well. It is perhaps of interest to remark, that the structure of such continuum damage mechanics models is different from the one according to ductile fracture models, for example, by Park and Kim [40], Papasidero et al. [41] or Cerik et al. [42]. A damage indicator variable is also used in these ductile fracture models, but this variable does not affect the elastic–plastic model responses. Of course, micromechanics damage models of the Gurson type (see, for example, Tvergaard and Needleman [43]) can also be incorporated, but such models do not account for damage degradation of the elasticity response as well, which is fundamental in the basic form of the model considered in this paper. A phase field theory for ductile materials of the proposed type will be discussed in forthcoming papers.

## Figures and Tables

**Table 1:** Material parameters used in the finite element model.

*K*	μ	$k_{0}$	γ=3c/2	$\left(1+\beta \right)G_{c}$	*l*
175,000MPa	80,769MPa	200MPa	5000MPa	2.7N/mm	0.0075mm

## Data Availability

Data sharing is not applicable to this article.
